# Squamous Cell Carcinoma of the Nail, an Underdiagnosed and Underestimated Entity: A Series of Two Cases

**DOI:** 10.7759/cureus.14826

**Published:** 2021-05-03

**Authors:** Camilo Levi Acuña Pinzon, Jefferson Fabian Nieves Condoy, Luis Abraham Zúñiga Vázquez, Gerardo Chavez Perez, Jose Luis Chavarría Chavira

**Affiliations:** 1 Surgery, Hospital Regional de Alta Especialidad del Bajío, León, MEX; 2 Surgery, Hospital Regional de Alta Especialidad del Bajio, León, MEX; 3 Surgical Oncology, Hospital Regional de Alta Especialidad del Bajío, León, MEX

**Keywords:** squamous cell carcinoma (scc), nail diseases, reconstructive flap surgery, nail cancer, moberg flap

## Abstract

The nail apparatus is a complex area with great functional and cosmetic importance. The appearance of tumors is rare, frequently misdiagnosed with delaying the diagnosis. A series of cases is presented, where squamous cell carcinoma of nail apparatus underwent resection and reconstructive surgery in a relatively short time from their diagnosis, with a good oncological, functional, and cosmetic result.

## Introduction

The nail apparatus is a complex area with great functional and cosmetic importance [1]. The appearance of tumors is rare but given the special anatomical location, they usually show morphological and clinical differences compared to similar lesions located in other parts of the skin [2], causing them to be frequently misdiagnosed as dermatoses, viral warts, or fungal infections, delaying the diagnosis on average five to seven years [3]. Despite being rare, squamous cell carcinoma of the nail unit is the most common malignancy of the fingertip [4].

We present a series of cases, where two patients with squamous cell carcinoma underwent resection and reconstructive surgery in a relatively short time from their diagnosis with a good oncological, functional, and cosmetic result.

## Case presentation

Case 1

A 69-year-old male was presented with no significant medical history, an active smoker with a smoking index of 26. Eight months ago he presented onycholysis in the first finger of his left hand, visiting an orthopedic specialist who performed an onychectomy. Six months later, he presented a recurrence of the lesion, for which he consulted again, doing this time a biopsy of tissue adjacent to onycholysis with a histopathological report of well-differentiated large cell squamous cell carcinoma. A plain oblique hand and dorsal-palmar X-ray is performed in which no cortical bone lesion is observed at the level of the distal phalanx.

**Figure 1:**
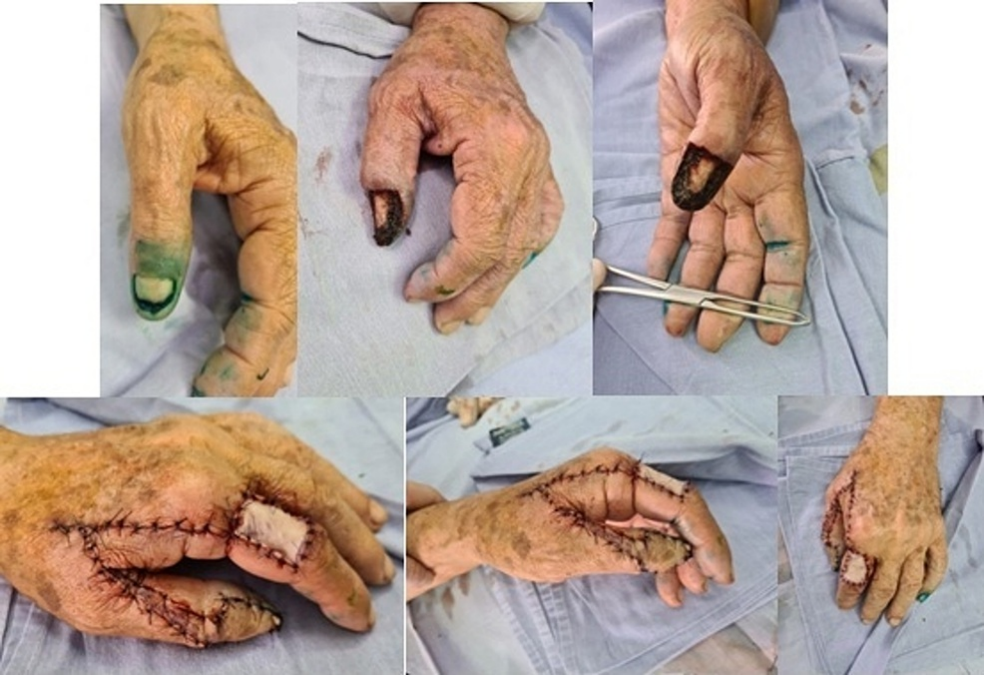
Radiopharmaceutical labeling, image post-resection, and final result. Top images correspond to radiopharmaceutical labeling in order to search for and resect sentinel lymph nodes, and a post-resection image of a lesion in the nail apparatus. Images below correspond to the final result after flap reconstruction of the first dorsal.

**Figure 2:**
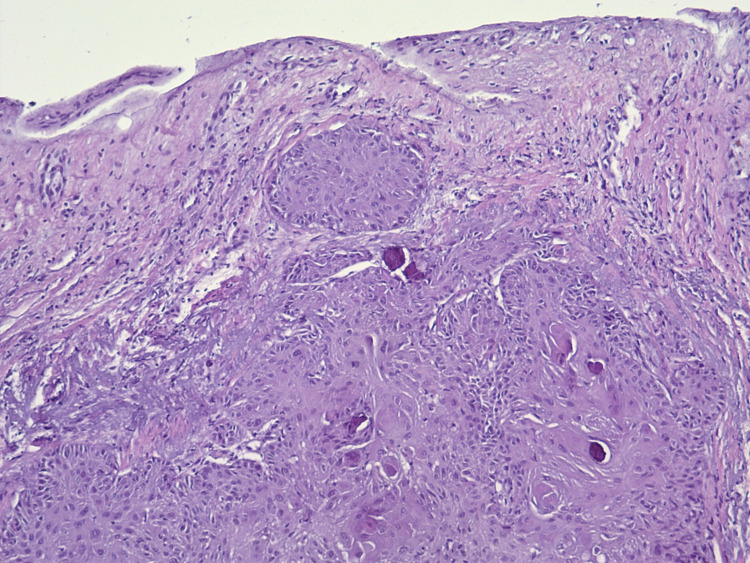
Well-differentiated squamous cell carcinoma, invasive keratinizing of the nail bed.

Twenty-six days later, he was taken to surgery, performing resection of the lesion, search for the sentinel lymph node with radiopharmaceutical, resecting the lesion and axillary lymph node (Figure 1). A surgical specimen was sent for intraoperative study, which reported lesion-free edges. Reconstruction with a flap of the first dorsal metacarpal branch of the second finger and placement of a left thigh graft (see Figure 1). Final histopathological report of surgical specimen informs well-differentiated squamous cell carcinoma, invasive keratinizing of the nail bed measuring 0.9 × 0.9 × 0.3 cm^3^ with resection margins free of neoplasia (Figure 2). Report of sentinel lymph node negative to malignidad.

**Figure 3:**
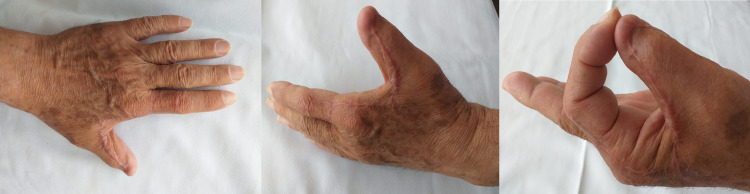
Control at seven months, the final result after reconstruction with a reverse Moberg flap.

Seven days after the surgical event under medical control, the suffering of the flap with partial loss was observed, which led to a surgical procedure again, this time carrying out dismantling of the previous flap and reconstruction with a reverse Moberg flap. There was good subsequent evolution, at seven months of follow-up without the presence of disease activity (Figure 3).

Case 2

**Figure 4:**
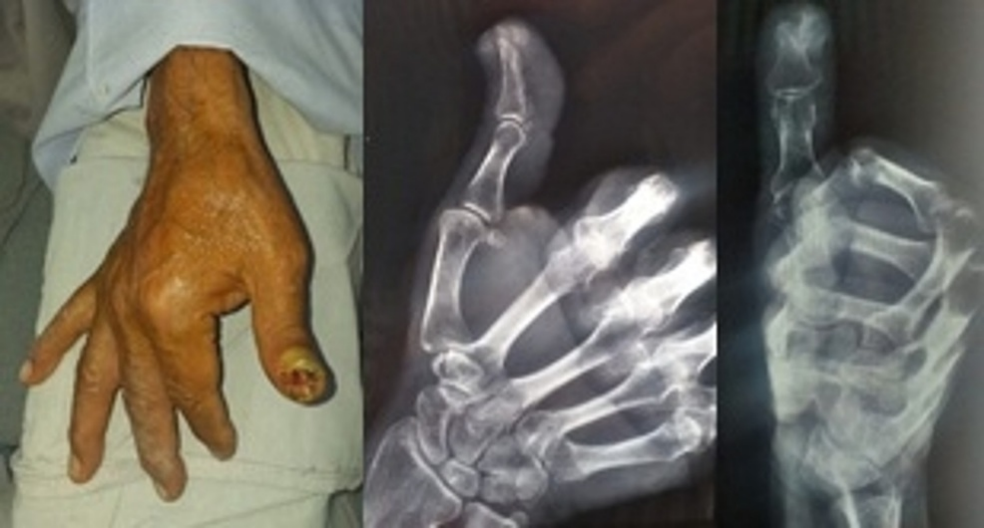
Tumor before surgery and osteolytic lesion in the distal phalanx of the first finger.

A 74-year-old male was presented with a history of systemic arterial hypertension under management with losartan and a smoking index of 15. Three years ago there was an appearance of a "line" on the nail of the first finger on the right hand, which subsequently presented pus. A year and a half later, they performed onisectomy without improvement, with a constant secretion that prevents adequate wound healing. Due to the lack of improvement, they performed a biopsy with a histopathological report of moderately differentiated invasive squamous cell carcinoma. Plain X-ray of the oblique hand and dorso-palmar was performed, showing an osteolytic lesion in the distal phalanx of the first finger (Figure 4).

**Figure 5:**
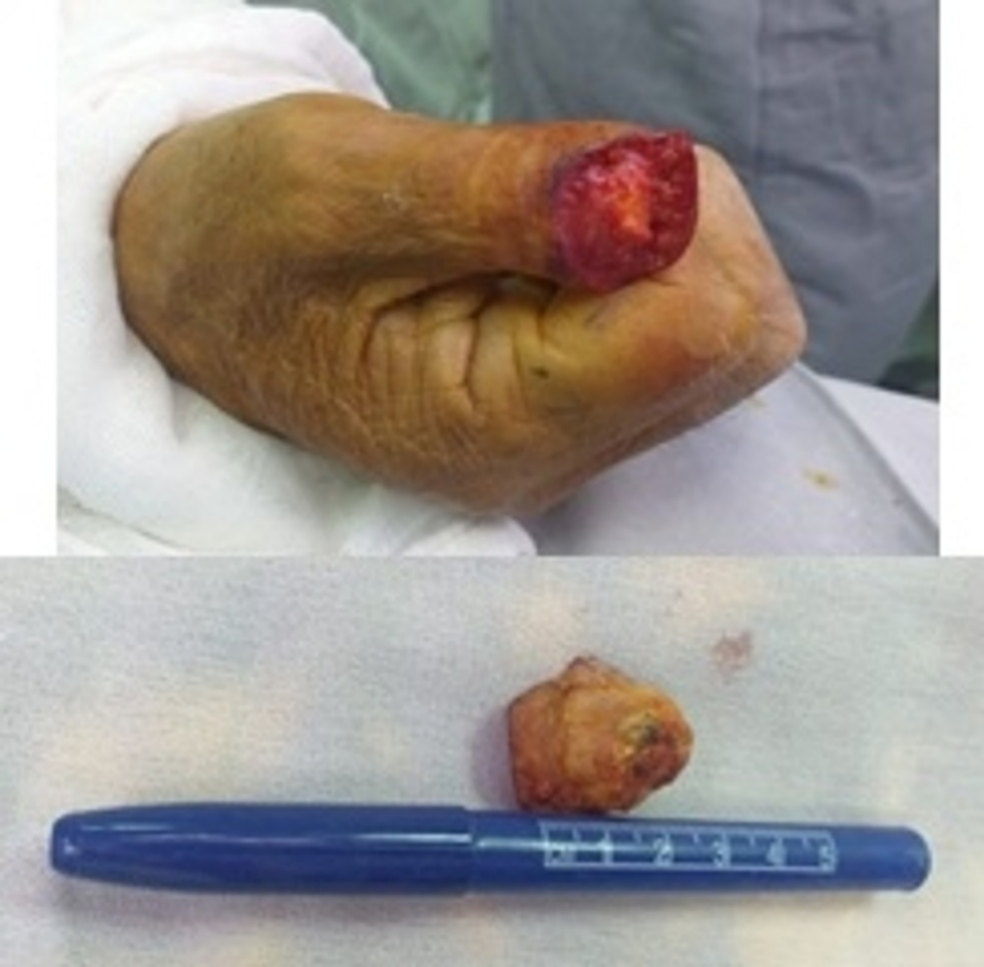
Resection of the lesion including periosteum.

**Figure 6:**
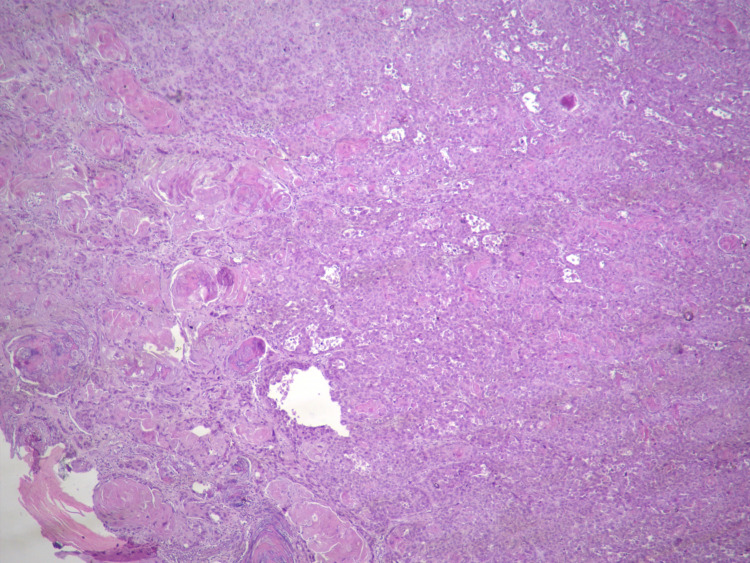
Well-differentiated invasive and ulcerated squamous cell carcinoma.

He was taken to surgery, performing resection of the lesion, with an incision at 1 cm from the tumor margin, extracting it to the periosteum (Figure 5). A piece of pathology is taken for intraoperative analysis that reports negative borders for malignancy; continuing with the reconstruction by means of a flap of the first dorsal metacarpal branch of the second finger and placement of a partial-thickness graft taken from the bicipital region + tie over. The final histopathological report of the surgical specimen reports a well-differentiated invasive and ulcerated squamous cell carcinoma of 1.5 cm; its major axis is associated with moderate chronic inflammation and mild desmoplasia with changes of mild necrosis, absence of vascular or neural permeation, periosteal infiltration, without evidence of activity on the edge of bone section (Figure 6).

**Figure 7:**
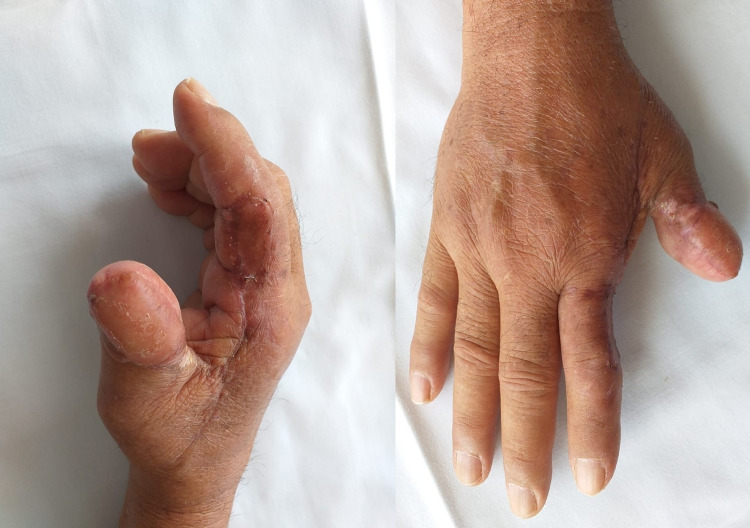
Control image two months post-surgical.

There was good evolution, at two months of follow-up without the presence of disease activity (Figure 7).

## Discussion

Squamous cell carcinoma is the second most common non-melanoma skin cancer, accounting for 20% to 50%. It usually presents as a red plaque in areas exposed to sunlight; these lesions being mostly solitary, although they can rarely present as multiple or "in-transit" metastases. Its diagnosis is made from a skin biopsy that must be deep enough to allow the pathologist to report the depth of the invasion, perineural or lymphovascular invasion, differentiation, and its connection with the underlying epidermis [5,6].

There are multiple histological variants that have been classified as low, moderate, or high-risk variants depending on their degree of differentiation (Table 1). In addition, rare variants have been described. The use of immunohistochemistry is not usually necessary, except in cases of poorly differentiated carcinoma or rare variants. Skin stains are positive with high molecular weight p63, p40, MUC1 (epithelial membrane antigen), CK5/6, MNF116, and 34 E12 [5].

**Table 1:** Histological variants of squamous cell carcinoma

Low and moderate risk	High risk	Infrequent variants
Keratoacanthomas	Acantolytic	Carcinoma with sarcomatoid differentiation
Verrucous carcinoma	Spindle cells	Lymphoepithelioma-like carcinoma
Clear cells	Adenosquamous carcinoma	Giant cell carcinoma

Squamous cell carcinoma can develop in different anatomical locations, among which are some high-risk locations such as the face, ears, pre-and post-auricular regions, genitalia, hands, and feet [7]. In the hands and specifically in the nail apparatus, the prevalence of squamous cell carcinoma ranges from 0.0012% to 0.028%, with a peak incidence between 50 and 69 years, despite the fact that the tumor can occur at any age. The incidence rate between men and women is 2:1. It usually involves only one finger, the thumb being the most frequent (44%) [7].

Within the etiopathogenesis, we find the development from predecessor lesions such as actinic keratosis, Bowen's disease, burn scars, and chronic radiation dermatitis [8,9]; the main risk factors are trauma, chronic exposure to sunlight or to arsenic, radiation, burns, genodermatosis, tobacco, immunosuppression, and human papillomavirus (HPV) infection. Immunosuppression plays an important role in tumor development since immunosuppressed patients initiate the disease at an earlier age and with a shorter history than patients with normal immune function [7].

Although 60% to 80% of cases have been reported to be associated with high-risk human papillomavirus, mainly serotype 16, which represents 57% of cases [9] (other serotypes that have been reported include 2, 6, 11, 18, 26, 31, 34, 35, 56, 58, 67 and 73) [10]. This fact has not been widely studied in systematic analyzes despite its great importance considering the higher recurrence rate, the possibility of metastasis, and the proliferative activity compared to HPV-negative carcinoma [7]. The possibility of digital-genital transmission and self-inoculation of HPV has been raised as part of its etiopathogenesis [9].

Usually, squamous cell carcinoma has an indolent course or causes very mild symptoms. In most cases, a long-standing lesion is described as a slow-growing tumor. Although the subungual region is the most frequent location, there is a great variety of clinical appearances depending on the region of the involved nail apparatus as follows [7]: (i) lateral detachment (Onycholysis) of the nail with a warty appearance of the exposed nail bed and the lateral nail fold that tends to ulcerate, associated with a thin adjacent band of longitudinal melanonychia, which results from melanocytic activation by the tumor. The warty appearance of the lesion explains why it is often misdiagnosed as a viral wart. (ii) Painless erosion of the nail bed, which appears as a distal area of onycholysis with yellow discoloration and suppuration under the nail with this erosion not visible unless the nail plate is cut. This clinical picture fits the patient in case 1. (iii) Great erosion of the nail bed associated or not with a nodule, usually observed in long-standing lesions. (iv) Longitudinal erythronychia band, clinically indistinguishable from erythronychia due to onychopapilloma, is rare and represents 3% of cases. This clinical picture fits the patient in case 2. (v) Carcinoma involving several fingers also known as synchronous squamous cell carcinoma. (vi) Hyperkeratosis in which the nail bed rises inducing onycholysis by parakeratotic accumulation.

Compared with squamous cell carcinoma of the rest of the skin, the one that involves the nail apparatus tends to be invasive more rapidly, such that bone involvement is observed in 16% to 66% of cases, with a higher incidence in immunocompromised patients [7]. According to Starace et al., pain is present only when there is bone invasion [7], however, as observed in case number 2, pain is not the main symptom of the patient.

As mentioned previously, the only way to confirm squamous cell carcinoma is by biopsy and it should be performed on each abnormal nail that does not respond to topical treatment. Different techniques for performing the biopsy have been described, but a nail bed biopsy is preferred once it has been exposed since a poorly planned biopsy will obtain an inadequate sample [11].

In the case of our patients, we do not know which technique was used to take the biopsy with which they were referred to consultation with oncological surgery; however, the pathological result of the same implies that they were taken properly.

Although the biopsy makes the definitive diagnosis, other elements can help or complement the study of a patient with squamous cell carcinoma. The use of nail dermoscopy (onicoscopy), a non-invasive method to better view the nail apparatus, has been shown to decrease the number of unnecessary resections. The alterations that are typically observed on onicoscopy are onycholysis, irregular vascularity, areas of hemorrhage with a rough verrucous surface. However, none of these signs are exclusive to the tumor, so despite improving the distinction between onychopapilloma, onychotrioma, subungual exostosis, and carcinoma, the differential diagnosis with periungual warts is not possible [7].

A recently used method is confocal fluorescence microscopy in which the finding of marked cytological and architectural atypia, nuclear pleomorphism, and densely clustered and irregularly organized nuclei correlate with invasive squamous cell carcinoma. Unfortunately, there is not as strong a correlation with minimally invasive carcinoma where mild cell atypia and "fuzzy cell pattern" are observed. In addition, its usefulness intraoperatively to make or confirm a diagnosis and to evaluate surgical margins has been suggested, being an alternative to classic Mohs surgery [7].

On ultrasound, a heterogeneous hypoechoic focal mass with irregular margins can be seen, which in the Doppler mode shows signs of low resistance pulsatile flow within the tumor or in its periphery [7]. The radiological examination should be performed to evaluate the bone compromise, which would be an indicator of amputation [11], however, this compromise is reported in less than 20% of the cases [7].

On tomography, it can be seen as a growing soft tissue mass with an osteolytic defect of the phalanx without periosteal reaction. A heterogeneous hypoechoic mass with an irregular contour and posterior acoustic enhancement is the most representative of carcinoma [7]. Chest tomography and an ultrasound looking for supratrochlear and axillary lymph nodes should be performed when metastatic disease is suspected on a routine basis [11] despite the fact that lymphatic involvement is less common, occurring in 2% of patients and metastases are even more infrequent [7].

Magnetic resonance imaging can be useful to detect a tumor when there is the presence of pain without clinical evidence of local lesion [11]; it is superior to other imaging methods due to its ability to accurately identify the location and extent of the tumor [7]; however, there are no studies that support MRI as the study of choice in addition to the difficulty in differentiating carcinoma from other conditions [11].

Unfortunately, at our institution, we do not have a nail dermatoscope or confocal fluorescence microscopy, for which reason these diagnostic tools were not used. Given the initial histopathological report and the use of radiography, it was not necessary to use other diagnostic means such as tomography or magnetic resonance imaging. In case 1, a complete resection associated with a search for a sentinel node was proposed to the patient, which in the absence of suspicion of metastatic disease made it unnecessary to carry out a chest tomography and ultrasound of tissues in search of lymphatic involvement. The treatment of choice is complete resection with an evaluation of the resection margins in three dimensions {1}, but this can be technically difficult given the anatomical complexity of the nail unit [11].

In case of bone invasion, amputation of the distal phalanx or disarticulation of the affected finger is recommended [1,11], being the latter treatment the one with the lowest recurrence rate [7]. In case 2, despite having bone involvement, opted for resection to the periosteum and not amputation gave the functional importance of the first finger (an opposite finger for clamp action). If there is no osseous invasion, a wide local resection should be performed, however, there is no clear information available on the optimal surgical margins [11]. A resection margin of no less than 4 mm has been recommended in some publications [7], however, other bibliographies recommend a margin of 5 mm [1].

Mohs micrographic surgery is indicated in non-invasive carcinoma allowing the resection of the diseased part of the nail apparatus with a minimal residual scar, reduced number of amputations, saving tissue, and preserving the quality of life of the patient with a better functional result. In addition, it allows evaluating periosteal invasion and distinguishes compression inflammation [7,11].

There are no case-control studies that compare the use of Mohs surgery with other techniques and rates of post-surgical complications in the treatment of squamous cell carcinoma of the nail apparatus, however, recurrence rates with Mohs surgery have been reported significantly lower [12]. In our institution, we do not have Mohs surgery or confocal fluorescence microscopy, for which it was decided to send the tissue to intraoperative study to define extending the resection based on its results, which was not necessary given the report of borders without tumor compromise.

Radiation therapy is useful in cases of the disease in multiple fingers or in which surgery is difficult, especially in immunocompromised patients, however, its benefits must be weighed against the risk of developing cancer from the same radiation, despite which has been suggested as a treatment to preserve the integrity of the distal phalanx [7,11].

An alternative and underused approach to surgical resection in difficult-to-treat cases is photodynamic therapy, a technique that is recommended in cases of extensive disease that, if treated with resection, would leave unacceptable functional or cosmetic sequelae; however, its use is limited due to its toxicity [7].

A variety of nonsurgical treatments have been tried with varying success rates including 5-fluoracil cream, imiquimod cream after curettage, cidofovir, and podophyllin. All these therapies share a high relapse rate and lack of histological control of the tumor margins [7,11]. Chemotherapy is indicated only in metastatic disease [7].

To perform a complete resection of the lesion and have a negative sentinel node report for malignancy, no other type of additional surgical or non-surgical treatment was necessary for the treatment of the tumor lesion, which confers a good prognosis for our patient.

Since a specific TNM classification for nails has not been developed to date, tumors are classified analogously to skin carcinomas [2]. Furthermore, there are no guidelines that advise a specific frequency or duration of follow-up, although some authors have suggested five years of follow-up and instruct patients to consult in case of any changes [11].

Once the complete resection of the lesion has been carried out, it is necessary to cover the defect generated when extracting the surgical piece, so reconstructive surgery is unavoidable. When selecting the best reconstructive option for fingertip injuries, three aspects should always be considered: the size of the defect, whether there is exposed bone or not, and the geometry of the injury [13].

The first dorsal metacarpal artery flap is one of the most used techniques for the coverage of defects in the proximal phalanx of the fingers, the first commissure, and especially for the reconstruction of the thumb, especially in extensive losses of substance from the dorsal surface of the first finger. It offers the advantages of being a sensitive flap, with a constant and safe vascular pedicle with multiple anastomoses that allow antegrade or retrograde designs, as well as the realization of compound flaps; it also produces little morbidity in the donor area and does not require the sacrifice of soft tissues in the adjacent fingers [14].

On the other hand, volar advancement flaps use neurotic skin with a volar characteristic and respect the functional aesthetic units of the thumb. The volar advancement flap described by Moberg is an advancement flap of the entire volar unit of the thumb that allows coverage of small to medium-sized lesions of the tip (up to 1.5 cm). Numerous modifications of the technique have been developed to increase advancements, such as performing bilateral Z-plasties, Burow's discharge triangles at the base of the thumb, or the proximal V-Y design described by Elliot. The V-Y advancement flaps (Atasoy) can be used in some cases of reconstruction of the thumb, although we generally use them for the reconstruction of long fingers of the hand [13].

We opted to initially perform a flap of the first dorsal metacarpal artery in order to cover the defect generated when resecting the lesion on the thumb of our patients, however, given the poor evolution in the first case, it was necessary to perform a reverse Moberg flap in a second surgical intervention after which I did not present new incidents and continued with good evolution. In both cases, there was a satisfactory cosmetic result and an acceptable functional one. Currently, patients continue in functional rehabilitation therapy of the thumb.

## Conclusions

Squamous cell carcinoma of the nail apparatus is a rare entity, which should be suspected in any nail lesion that does not respond to topical treatment. The mainstay of treatment continues to be resection with margins without tumor involvement. There is still no consensus on the resection margins and their follow-up. Complete removal of the lesion plus the periosteum of the compromised phalanx may be a treatment for patients with bone invasion; however, more studies are required to make a recommendation. Reconstruction of the defect left by resection of the tumor is of great importance given its cosmetic and functional impact.
